# A retrospective study to understand the differences in maternal mortality among women admitted in critical and stable conditions in Malawi

**DOI:** 10.1136/bmjph-2024-001172

**Published:** 2025-01-19

**Authors:** Laura Munthali, James Chirombo, Lumbani Makhaza, Jennifer Riches, Malangizo Mbewe, Rosemary Bilesi, Nara Tagiyeva-Milne, David Lissauer

**Affiliations:** 1 Liverpool School of Tropical Medicine, Liverpool, UK; 2 Malawi-Liverpool-Wellcome Trust Clinical Research Programme, Blantyre, Malawi; 3 Women's and Children's Health, Institute of Life Course and Medical Sciences, University of Liverpool, Liverpool, UK; 4 Ministry of Health, Reproductive Health and Quality Management Directorates, Lilongwe, Malawi

**Keywords:** Public Health, Epidemiology, Public Health Practice

## Abstract

**Introduction:**

Addressing the burden of preventable maternal deaths remains a critical focus of global health efforts in countries like Malawi which still has a high maternal mortality ratio of 381 deaths per 100 000 live births. We investigated the differences in case characteristics, survival and causes of death between pregnant and recently pregnant women who died, following their admission in stable or critical conditions to healthcare facilities in Malawi.

**Methods:**

We conducted a retrospective analysis of maternal deaths of women from all district and central hospitals of Malawi between August 2020 and August 2022. Data were sourced from the national maternal and newborn health surveillance platform. We performed descriptive analyses, stratified by condition on admission, to identify differences in case characteristics between the two groups, and an exploratory survival analysis, to understand the differences in time to death since admission. Lastly, we performed a principal component analysis to reduce dimensionality to identify the main factors leading to deaths.

**Results:**

Obstetric haemorrhage was the predominant cause of death among women admitted in stable condition, while those admitted in critical condition primarily died of hypertensive disorders. Most deaths in both categories occurred on the day of admission, corresponding to their primary causes of death. The key factors leading to deaths were mostly healthcare worker factors followed by administrative factors.

**Conclusion:**

Understanding and responding to the different underlying causes of maternal mortality and contributing factors in the stable and critical cohorts are vital to designing well-targeted and impactful interventions to prevent maternal deaths.

**WHAT IS ALREADY KNOWN ON THIS TOPIC**

- Despite extensive research on women in critical condition, little attention has been given to those admitted in stable condition, who also contribute significantly to maternal mortality. In Malawi, data show that women in stable condition also play a major role in maternal deaths, yet there is no universal consensus on how to define and assess stable versus critical conditions.

**WHAT THIS STUDY ADDS**

- The study calls for standardised protocols for assessing women at admission to reduce preventable deaths, particularly in low-resource settings. It stresses the importance of improving healthcare worker competency and developing clear tools for defining admission status, as current assessments are often subjective.

**HOW THIS STUDY MIGHT AFFECT RESEARCH, PRACTICE OR POLICY**

- The findings advocate for policy changes including targeted interventions that ensure comprehensive care for women admitted in both stable and critical conditions to improve maternal health outcomes worldwide.

## Introduction

Addressing the burden of preventable maternal deaths has been a prominent focus of global health efforts for a prolonged period. The establishment of Millennium Development Goal (MDG) 5a (75% reduction of global Maternal Mortality Ratio (MMR) from 1990 to 2015) in 2000 reflected a global commitment to ending preventable maternal deaths.^1^ Despite these efforts, the goal remained unmet, with Sub-Saharan Africa (SSA) significantly contributing to the deaths. In 2017, the region accounted for 66% of the estimated 295 000 global maternal deaths.^2^ The global commitment to decrease maternal mortality is evident in the current Sustainable Development Goal (SDG) 3.1, which aim to lower maternal deaths to less than 70 per 100 000 by 2030.^2 3^

Although Malawi has made notable progress in reducing maternal deaths, it still struggles with a high MMR of 381 deaths per 100 000 live births.^4^ To address the high mortality burden and improve the quality of care during pregnancy, delivery and the postpartum phase, Malawi introduced the National Maternal and Newborn Health Surveillance Digital (MATSurvey) platform in July 2020. The platform was established to collect real time data on the effects of emerging diseases on maternal and newborn outcomes, near miss cases, maternal deaths and quality of care indicators.^5^ MATSurvey data revealed that women admitted to the hospital in both stable and critical conditions contribute significantly to maternal deaths^6^ an unexpected finding, as such deaths could be anticipated among those admitted in critical condition. The World Health Organisation (WHO) states that the majority of maternal deaths are preventable through timely interventions and proper care, though some remain non-preventable even with optimal treatment^2 7^

Few studies have been conducted to compare the condition in which pregnant or recently pregnant women were admitted, whether critical or stable, and the sequence of events that resulted in their death globally. The condition at which women are admitted plays a vital role in shaping the type of care they receive and influencing the outcomes. However, there has been no global consensus in describing the conditions stable or critically ill on admission as the terms have been subjective and vary depending on the health facility, healthcare provider experience, context and region leading to differences in management, care as well as outcomes.^8 9^ Some describe critical illness based on organ dysfunction observed through vital signs and tests, while others associate it with Intensive Care Unit admission following abnormal observations.^10 11^ Despite these differences, there is consensus that critical illness involves a high risk of death due to organ failure, requiring urgent care.^11^ Admission in a critical condition has often been linked to poor outcomes, especially within the first 24 hours.^12^ In High-Income Countries (HICs), tools like the Early Obstetric Warning chart help assess stability, whereas in Low and Middle-Income Countries (LMICs), clinical judgement is mostly relied on due to a lack of such tools.^13^ To be considered stable, the woman must have normal observations and assessment scores conducted by healthcare providers; however, in resource constraint settings, this can be compromised leading to worst outcomes.^13^ However, in some instances, stable has been used to describe both critically ill and non-ill patients which provides a huge gap in classification as well as care provision.^14^ In Malawi, while the Malawi Standard Treatment Guidelines provide guidance for medical practice, they do not offer clear understanding for stable or critical conditions in maternal health.^15^ It is with this background that this study aimed to investigate the differences in the demographic, case characteristics, causes of death and time to death between women admitted in stable and critical conditions between August 2020 and August 2022. Further research is essential in SSA to better understand the admission conditions of recently pregnant women. Such studies are crucial in provision of valuable insights for developing targeted interventions aimed at improving care and management to reduce maternal mortality.

## Methods

### Study design and setting

This was a retrospective analysis of prospectively collected data from the MATSurvey platform. The MATSurvey platform collects data at the district and central hospital levels. This study included maternity units across all 28 secondary district hospitals and four central tertiary hospitals of Malawi, primary healthcare facilities, including community or rural hospitals, private hospitals, and those affiliated with the Christian Health Association of Malawi (CHAM), and direct emergency cases to district hospitals for specialised care. District hospitals, in turn, handle referrals to central hospitals when necessary. The conduct of this study adhered to Strengthening the Reporting of Observational Studies in Epidemiology (STROBE) statement available in the online supplemental material.

### Data source

This study used data from Malawi’s MATSurvey platform, a component of the National Maternal and Perinatal Death Surveillance and Response (MPDSR) system. While Malawi traditionally relied on the District Health Information System (DHIS) 2 for collecting monthly aggregated maternal and perinatal death data, the continued use of paper-based forms in many LMICs, including Malawi, has raised concerns about data accuracy and reliability.^16^ To address the issue, digital systems have been recommended for real-time data collection from the point of care.^16^,^18^ In response to the COVID-19 pandemic in 2020, Malawi established the MATSurvey platform to track the pandemic’s direct and indirect impacts on maternal and newborn health. The platform also digitalised essential forms (Maternal Death Audit (MDA) forms 1 and 2, and near-miss reporting) and quality of care indicators, enhancing real-time reporting of maternal deaths. Unlike DHIS 2, which aggregates data monthly, MATSurvey provides detailed, disaggregated reports, along with weekly data on maternal and newborn outcomes and care quality. On maternal death entry into the platform, each woman was assigned an admission status by the hospital staff, categorised as stable, critical or dead on arrival, in accordance with MPDSR guidelines. The platform’s dashboard supports decision-making at all levels, with the Ministry of Health (MoH) ensuring data confidentiality and secure access based on ethical standards. All deaths analysed in the study from the platform were audited by district MPDSR committees.

### Population and data extraction

The population in the study comprised cases of women who died while pregnant or within 42 days of delivery or termination of pregnancy. The data covered the period from August 2020 to August 2022 with 702 audited records of maternal deaths. A total of 660 maternal deaths of women admitted in stable and critical conditions were included for analysis. Deceased women, 42, brought into the facilities were excluded from the analysis. Data on demographic and case characteristics, causes of death and factors associated with the deaths were extracted from individual MDA forms. Total live births data were derived from the weekly aggregated forms. Causes of death were classified according to the WHO International Classification of Diseases Maternal Mortality.^19^ A final dataset for the analysis was created by merging the described data sources. However, there were some missing data on some of the variables in the data.

### Sampling and sample size calculation

A formal sample size calculation was not performed for this study. The MATSurvey platform captures data from all districts in Malawi, ensuring comprehensive geographical coverage and including various demographic and case characteristics. Maternal deaths are infrequent occurrences, and attempting to obtain a sample within this specific period could have resulted in a small sample size, thereby reducing the statistical power of the study.

We included all the data from all the districts in Malawi in our analysis to have good representation across all the districts and between urban and rural. This was done to minimise possible bias that may arise due to underrepresentation of some sections of the society.

### Statistical analysis

We performed descriptive analysis, stratified by condition on admission, whether stable or critical. Tables of demographic and case characteristics and causes of death, all stratified by condition on admission, were generated. Categorical variables were summarised using proportions, while continuous variables were summarised using medians and IQR. Comparison of the median values of numeric variables, such as age, and gravidity, between stable and critical groups was done using the Wilcoxon rank sum test. We also used Fisher’s exact test to test for the association between the condition on admission and key categorical variables. All statistical tests were done at the 5% significance level.

Trends in MMR, given by total maternal deaths/total live births × 100 000 were calculated and presented using a line graph. We then carried out a survival analysis to explore the time duration from admission to death stratified by causes of maternal deaths. Kaplan-Meier curves were constructed for both stable and critically ill women to visualise differences in survival times. A formal test to compare the difference in survival time by cause of death was done using the log rank test.

We also conducted a Principal Component Analysis (PCA) to reduce the dimensionality of the factors associated with maternal deaths. The PCA was used to find the best low dimensional representation of variation in the data and to determine which variables contributed strongly to the various principal components. We retained principal components that cumulatively accounted for at least 50% of the variation. We then selected the variables that contributed the most to each of the retained principal components. Assuming equal contribution, each variable contributes 2.55%. However, to obtain variables with a stronger influence on the components, we chose variables that contributed at least 4% to all components. We used the selected variables to further understand their collective contribution to the reported maternal deaths in the two groups of women.

## Results

### Demographic characteristics

Overall, 660 women were admitted in stable or critical conditions during the study phase. table 1 illustrates their demographic characteristics. There was an observed difference in parity, gravidity, gestation and admitted from between the stable and critical groups (p<0.01). However, there was no difference in age, marital status and education between the two groups. The majority of women admitted in critical condition were admitted from another facility as compared with women admitted in stable condition, who were mostly admitted from their homes.

**Table 1:** Demographic characteristics of women admitted in stable and critical conditions who died from August 2020 to August 2022

	Stable (n=262)	Critically ill (n=398)	Total (n=660)	P value*
Age (years)				0.54
N	261	398	659	
< 19	28 (10.7%)	52 (13.1%)	80 (12.1%)	
20–24	42 (16.1%)	71 (17.8%)	113 (17.1%)	
25–29	39 (14.9%)	57 (14.3%)	96 (14.6%)	
30–34	44 (16.9%)	53 (13.3%)	97 (14.7%)	
35–39	35 (13.4%)	41 (10.3%)	76 (11.5%)	
40+	73 (28.0%)	124 (31.2%)	197 (29.9%)	
Marital status				0.69
N	239	357	596	
Single	20 (8.4%)	39 (10.9%)	59 (9.9%)	
Married	214 (89.5%)	310 (86.8%)	524 (87.9%)	
Divorced	4 (1.7%)	5 (1.4%)	9 (1.5%)	
Widowed	0 (0.0%)	2 (0.6%)	2 (0.3%)	
Separated	1 (0.4%)	1 (0.3%)	2 (0.3%)	
Education				0.54
N	238	346	584	
None	68 (28.6%)	118 (34.1%)	186 (31.8%)	
Primary	120 (50.4%)	163 (47.1%)	283 (48.5%)	
Secondary	40 (16.8%)	53 (15.3%)	93 (15.9%)	
Higher	10 (4.2%)	12 (3.5%)	22 (3.8%)	
Admitted from				< 0.01
N	262	398	660	
Another facility	106 (40.5%)	294 (73.9%)	400 (60.6%)	
Community	4 (1.5%)	4 (1.0%)	8 (1.2%)	
Home	140 (53.4%)	94 (23.6%)	234 (35.5%)	
Other	12 (4.6%)	6 (1.5%)	18 (2.7%)	
Parity				0.02
N	250	351	601	
0–1	75 (30.0%)	149 (42.5%)	224 (37.3%)	
2–3	90 (36.0%)	105 (29.9%)	195 (32.0%)	
4–5	61 (24.4%)	71 (20.2%)	132 (22.0%)	
6+	24 (9.6%)	26 (7.4%)	50 (8.3%)	
Gravidity				0.02
N	249	341	590	
0–1	56 (22.5%)	113 (33.1%)	169 (28.6%)	
2–3	93 (37.3%)	107 (31.4%)	200 (33.9%)	
4–5	60 (24.1%)	80 (23.5%)	140 (23.7%)	
6+	40 (16.1%)	41 (12.0%)	81 (13.7%)	
Gestation				< 0.01
N	246	313	559	
Median (IQR)	38.0 (36.0, 38.0)	36.0 (30.0, 38.0)	37.0 (32.0, 38.0)	

* p Values for the difference in all the categorical variables between stable and critical groups based on Fisher’s exact test. For gestation, p value is based on the Wilcoxon rank sum test.

### Case characteristics

The antenatal care attendance, the use of partographs and having delivered before death were significantly more common among women admitted in stable condition compared with those in critical condition (p<0.01) (online supplemental table S1 in the supplementary materials). There were also differences between the two groups in the mode of delivery, the healthcare worker who delivered the baby and conditions at death (p<0.001). Compared with women in critical condition, spontaneous vaginal deliveries were less common, and caesarean section was more common among women in stable condition. Clinical officers conducted most deliveries of women admitted in stable condition compared with women who were admitted in critical condition whose deliveries were mostly conducted by midwives (p<0.01). It was observed that a higher proportion of women who were admitted in stable condition died within the immediate postpartum phase (within 24 hours) compared with those who were critically ill on admission. There was no statistical difference between the two groups over danger signs, where labour started, and the ownership of the health facility where the deaths occurred.

### Changes in maternal mortality ratio (MMR) in Malawi over time

**Figure 1:**
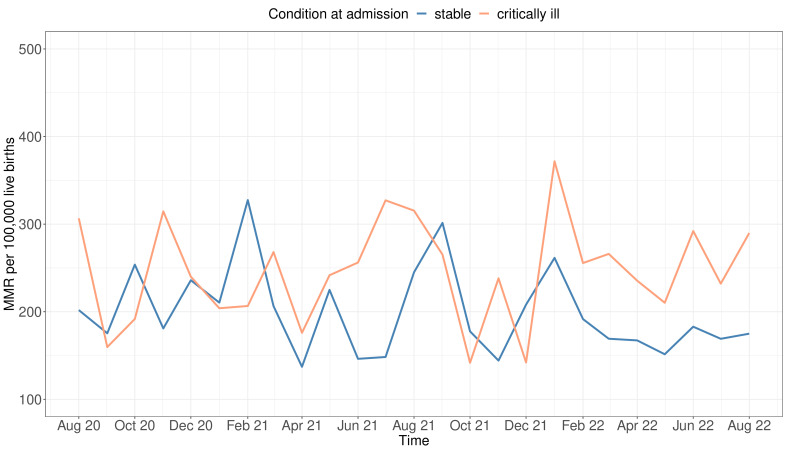
Temporal changes in maternal mortality ratio (MMR) of women admitted in critical and stable condition over the study period between August 2020 to August 2022.

Figure 1 depicts the temporal fluctuations in MMR between the two groups throughout the study period. The peak MMR occurred among women admitted in critical condition, surpassing 350 deaths per 100 000 live births in January 2022. Generally, MMR consistently remained higher for women in critical condition, except for September and October 2020, as well as January, February, September and October 2021. For women admitted in stable condition, the highest MMR was observed in February and September 2021. Temporal changes according to individual districts are illustrated in the supplementary materials online supplemental figure S1.

### Causes of maternal deaths

**Figure 2:**
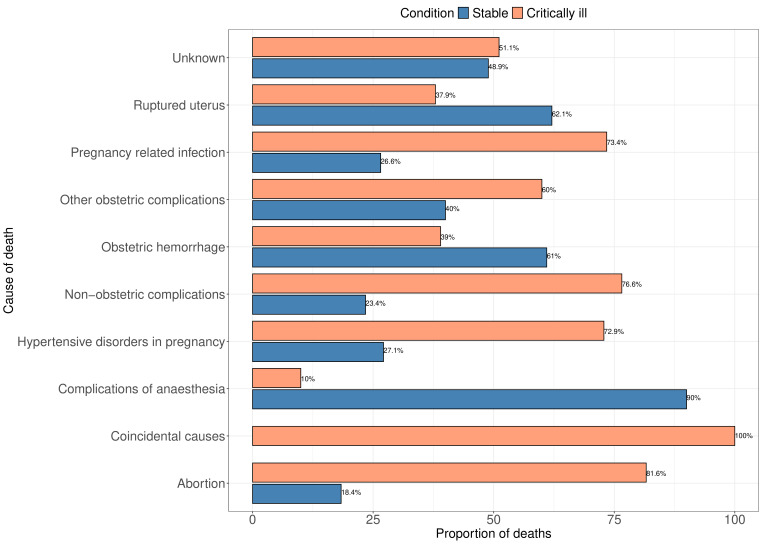
Proportion of maternal deaths among women admitted in stable and critical conditions due to different causes.

Overall, the leading causes of maternal deaths were obstetric haemorrhage, hypertensive disorders in pregnancy and non-obstetric complications which caused 24.2%, 21.3% and 16.9% of all maternal deaths. When stratified by condition at admission, there were notable differences as shown in figure 2. Among women admitted in stable condition, complications of anaethesia, ruptured uterus, obstetric haemorrhage and hypertensive disorders in pregnancy accounted for the highest proportions of deaths at 90%%, 62.1%, 61% and 27.1%, respectively. Among women admitted in critical condition, the leading causes of death were abortion, non-obstetric complications, pregnancy-related infection, hypertensive disorders and other obstetric complications accounting for 81.6%, 76.6%, 73.4%, 72.9% and 60% of the deaths, respectively.

### Time to death of women admitted in critical and stable condition by cause of death

In the stable group, 29% of women died on admission day, 24% died on day 1, 10.7% on day 2, 5.3% on day 3 and 5.0% on day 4, while 26% died on day 5 and beyond. In the critically ill group, 38.9% died on the day of admission, 21.4% on day 1, 7.29% on day 2, 4.27% on days 3 and 4, and 23.9% on day 5 and above.

**Figure 3:**
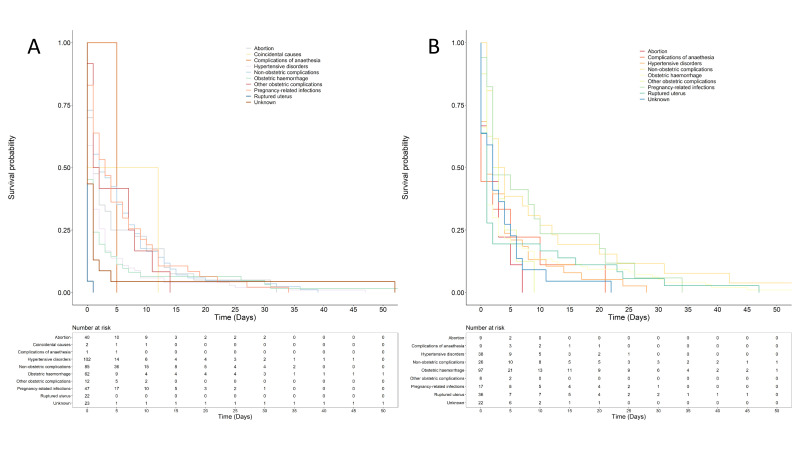
Kaplan-Meier curves indicating time to death due to the leading causes of death (**A**) critical group and (**B**) stable group.

Figure 3A and B show the survival of women admitted in critical and stable conditions, respectively, stratified by cause of death. There was a statistically significant difference in the length of time from admission to death due to various causes (p<0.01). Notably, across all causes of death, over 50% of women admitted in critical or stable condition had died by day 5. Across specific causes of death, women in critical condition and admitted with a ruptured uterus had the shortest survival time, with all deaths occurring before day 5. Hypertensive disorders emerged as a significant contributor to mortality, with only 14 out of 102 women admitted in critical condition being alive on day 5. In general, there were relatively longer survival times in the stable group compared with the critical group.

### Associated factors leading to maternal deaths

From the PCA results, we selected the first five principal components that cumulatively explained 50% of the total variation in the mean number of monthly maternal deaths reported among all women due to various causes (see online supplemental figure S2 in the supplementary materials). We then selected the variables that contributed the most to each of the retained five principal components. Broadly, PC1 mainly capture healthcare worker factors, while PC2 and PC3 represent patient-level/community factors. PC4 and PC5 mainly capture a combination of administrative, community/family and patient level factors (see online supplemental table S2 in the supplementary materials). The selected variables that contributed at least 4% to each of the selected principal components are shown in online supplemental table S3 in the supplementary materials.

**Figure 4:**
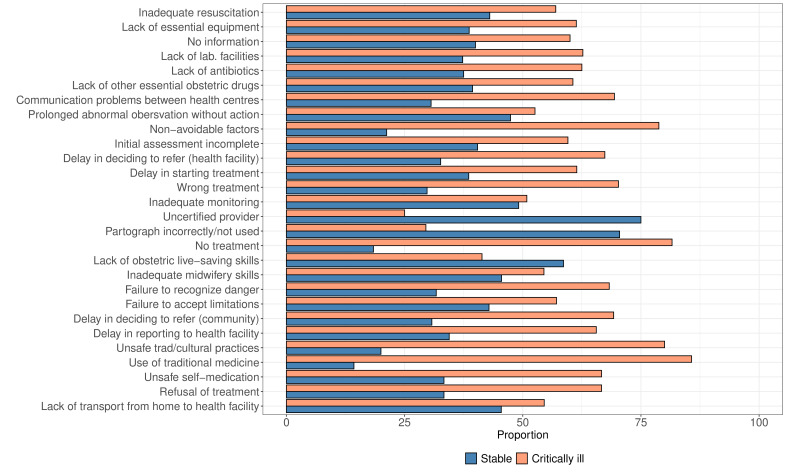
The proportion of maternal deaths among women admitted in stable and critical conditions attributed to each associated factor leading to mortality.

The proportion of deaths attributed to each factor is shown in figure 4. Higher proportions of deaths due to leading causes of death such as delayed treatment (61.4%), prolonged observation (52.6%), incomplete assessment (59.6%), delayed referral (65.6%), lack of equipment (61.3%), inadequate monitoring (50.9%) and inadequate resuscitation (57.0%) were observed among women who were admitted in critical condition compared with those who were admitted in stable condition.

## Discussion

To the best of our knowledge, studies looking at maternal mortality have mainly focused on all women as a single group; little focus is given to the condition in which the women were admitted before their deaths. By stratifying into stable and critical groups, we showed that key differences exist in the case characteristics, survival and causes of death.

The findings about the leading causes of maternal deaths being obstetric haemorrhage, hypertensive disorders in pregnancy, non-obstetric complications (indirect causes) and pregnancy-related infections support global patterns which rank obstetric haemorrhage as the leading direct cause of maternal mortality globally followed by hypertensive disorders and sepsis.^7^ According to the International Classification of Diseases, Tenth Revision, there are nine groups classified as causes of maternal deaths including pregnancies with abortive outcomes; hypertensive disorders in pregnancy, childbirth and the postpartum period; obstetric haemorrhage; pregnancy-related infection; other obstetric complications; unanticipated complications of management; non-obstetric complications; and unknown and coincidental causes.^19 20^ These categories are further divided into subcategories. Our study analysed the main categories, thereby providing insight towards further investigations of the subcategories for targeted interventions for women admitted in stable and critical conditions to promote maternal health. Interestingly, a relatively high proportion of deaths due to obstetric haemorrhage were from women admitted in stable condition. On the other hand, a high proportion of women admitted in critical condition died of hypertensive disorders in pregnancy. These results highlight the healthcare challenges encountered in low resource settings. The results indicate the need for focused interventions on admission. Women admitted in stable condition are less likely to face mortality when adequate management and care are provided. This is also true for those in critical condition who died mostly of hypertensive disorders, especially when nations have adopted and implemented guidelines for hypertension management and care of pregnant women.^21^ Complications may have developed for women admitted in stable condition within the facilities which resulted in their death.^22^,^27^

Many studies have indicated that most maternal deaths in low resource settings occur within the first 24 hours after admission.^23^,^25^ However, most of these studies did not specify the condition at which women were admitted and their length of stay in the hospital. We found that in both groups of women, there was a significant occurrence of mortality within the first 24 hours and a few days following admission. The exact timing of death was dependent on the underlying causes of death. However, it was clear that the three leading causes of maternal deaths (obstetric haemorrhage, hypertensive disorders and infections) and non-obstetric causes resulted in the death of more women however being different according to the condition at admission. Outlining interventions specific for managing women from the point of admission based on their condition of admission is essential to reduce mortality. Our study indicates that point of admission care of women in maternity settings and management are critical for achieving good outcomes, particularly because the early period following admission is also the frequent timeframe for women’s mortality. With lack of full assessment and care at admission, most deaths occur within a few hours of admission from preventable causes. In Malawi, poor hospital care and lack of adequate resources are some of the challenges that have been documented leading to poor maternal outcomes.^2226^,^28^ Our research aligns with previous studies advocating the significance of the admission day in averting maternal deaths within low resource settings. The imperative for devising strategies that enhance admission interventions and prioritise resource allocation is underscored as crucial in the prevention of maternal mortality^29 30^

Our PCA results showed the importance of healthcare workers and administrative factors in contributing highly towards the deaths for both groups. Delayed treatment, prolonged observation without action and incomplete assessment were some of the healthcare worker factors that were highly cited in our study to have led to the death of women in both groups, despite being more prominent in the critically ill group. Other studies describing factors associated with maternal mortality have described healthcare worker factors as being associated highly with maternal mortality.^31^ Inadequate human and material resources and congested facilities have also been cited as reasons for compromised provision of quality care hence poor outcomes.^25^ In Malawi, healthcare facilities, especially district hospitals, have low numbers of doctors, nurses and clinical officers on duty versus the number of hospital admissions.^28^ This is likely to lead to poor management of women at admission and during hospital stay leading to poor outcomes. Maternal outcomes would improve if appropriate, and adequate expertise is provided for in the health facilities, and strict supervision over the management and care of women was enforced at all levels. Other causes of death such as delay in reporting to health facility highlight the critical role that community-based interventions can play in ensuring good maternal outcomes.

Despite over half of the deaths of women admitted in stable condition admitted from home, a significant proportion of above 40% were referred from another facility. The majority of deaths of women admitted in critical condition were among those that were referred from another facility. These results suggest poor condition and prognosis from admission and throughout stay in hospital especially for women admitted in critical condition. Another study conducted in Zambia revealed that although 90% of referral facilities had professional healthcare providers, there were limitations such as a shortage of skilled personnel, lack of referral standards for complications, disruptions in the referral network, transportation issues and a lack of feedback mechanisms, thereby resulting into poor health outcomes.^32^ A study investigating factors associated with mortality in Malawi equally highlighted delay in the referral of maternal cases between health facilities as a contributing factor to maternal deaths.^31^ These observations agree with our PCA results which equally indicated delay in deciding to refer and lack of transport as some of the main factors associated with mortality (see figure 4).

Our findings revealed no difference in the common age of death between the groups as the majority of deaths occurred among women above 40 years. However, relatively higher mortality among women aged 20 to 24 for women admitted critically ill compared with those admitted in stable condition in the same age band was observed. This finding is particularly noteworthy, especially considering that certain studies suggested that adolescent women were more likely to die at health facilities compared with older women.^3133^,^35^ The variations observed may be attributed to our study analysis which focused on maternal deaths which were reviewed across all district and central hospitals in Malawi with the exclusion of those who were already dead on arrival. Recent studies have indicated a higher risk of mortality in older age groups^33^ which are similar to our findings for women who were admitted in stable and critical condition before their death.

We found higher caesarian section rates among women admitted in stable condition compared with those in critical condition who predominantly had spontaneous vaginal deliveries. Although caesarian section rates have increased globally, there have been no reported maternal and perinatal benefits.^36 37^ Caesarean section rates are higher in HICs compared with LMICs where resource limitations and infrastructure may affect the availability and preference for the procedure.^38 39^ In Malawi, institutional deliveries increased from 55% in 1992 to 91% in 2015/2016 including caesarian sections from 3% to 6% in the same time frame.^40^ Our study could not justify the reason for higher caesarean sections among women admitted in stable condition because of lack of specificity of whether the procedure was planned or unplanned in the audit forms. However, more studies to understand the increase in caesarian sections in low resource settings would be necessary.

These findings support the strategies and actions for improving maternal health and reducing maternal mortality and mortality^41^ as well as the WHO recommendations on health promotion interventions for maternal and newborn health which aim to improve the health of women and reduce preventable maternal deaths. The findings highlight the importance of targeted interventions in the management of women admitted to maternal care units, whether in critical or stable condition. This study indicates that there is little difference in the MMR between the two groups, underscoring the need for tailored approaches to care in both scenarios. However, our discoveries contribute a significant facet aligning with global health initiatives striving to decrease maternal deaths by 2030, especially in SSA where maternal deaths are exceptionally high^2^

### Strengths and limitations

The analysis included all audited deaths of women who were admitted in stable and critical conditions before death recorded on the MATSurvey platform with prospectively collected data of a wide coverage across multiple institutions and a range of factors available for the analysis. This aligns with the global objective to reduce preventable maternal deaths, which remains a significant concern.^3 27^ The stratified analysis by condition on admission is equally the major strength of this work. It highlights the equally high rates of mortality among women who were admitted in stable conditions and therefore should have survived. The study also reveals the shortcomings at different levels that should be acted on to reverse the current trends in maternal mortality. One key limitation is that MDAs face inconsistent implementation, with irregularities in timely audits even when reporting is conducted.^42^ This variability affects result accuracy, particularly when districts show high or low MMRs based on facility audits. Another limitation was the scope of analysis regarding the causes of death, which did not include the subcategories within each main cause. Further research into these subcategories is needed to develop targeted interventions for each specific group. Additionally, the study exclusively analysed maternal deaths of women who were admitted in stable and critical conditions, lacking information on women in similar conditions who survived and died earlier. Our study period of 2 years was also relatively short. A longer time series of maternal deaths would have provided more insight into the differences in outcomes between the two groups. The PCA also explained relatively little variation in the data. For this reason, the results of the associated factors leading to mortality are exploratory. A more comprehensive community and hospital-based study would be necessary to confirm our results about the factors leading to death and qualitative research to understand barriers to access of care and unmet needs.

## Conclusion

In this study, we were able to reveal hidden patterns within the two cohorts. The study provides valuable insights for healthcare administration and management globally to achieve SDG of less than 70 deaths per 100 000 live births by 2030. The study emphasises notable differences in demographics, case characteristics, causes and timing of death, and contributing factors between two cohorts of women admitted in critical and stable condition before their death. These insights are essential for informing global and national policies, as well as strategies to prevent maternal deaths and improve healthcare systems and quality of care from the point of admission. Conducting additional research on women admitted in stable or critical conditions and analysing their outcomes could provide further insights, ultimately contributing to the refinement of policies, standards, and funding in this area.

## Supplementary material

10.1136/bmjph-2024-001172 online supplemental file 1

## Data Availability

Data are available upon reasonable request.
