# Correction: A retrospective study to understand the differences in maternal mortality among women admitted in critical and stable conditions in Malawi

**DOI:** 10.1136/bmjph-2024-001172corr1

**Published:** 2025-12-02

 Munthali L, Chirombo J, Makhaza L, *et al.* A retrospective study to understand the differences in maternal mortality among women admitted in critical and stable conditions in Malawi. *BMJ Public Health* 2025;3:e001172. https://doi.org/10.1136/bmjph-2024-001172.

This article has been corrected since it was first published. The following award was missing from the funding statement and has now been added: NIHR134781 Improving the quality of maternal healthcare in Africa.

