# Supplementary material for: A retrospective study to understand the differences in maternal mortality among women admitted in critical and stable conditions in Malawi
Source: BMJ Public Health. 2025 Jan 19;3(1):e001172. doi: 10.1136/bmjph-2024-001172 (PMC11816890; doi:10.1136/bmjph-2024-001172)
Supplement: online supplemental file 1 [file bmjph-3-1-s001.pdf]

Supplementary materials

Table of Contents

Case characteristics of women admitted in stable and critical conditions 1

Temporal changes in maternal mortality at the district level 2

Principal component analysis 3

Selection of variables with a stronger contribution to principal components 4

STROBE Statement—Checklist of items that should be included in reports of *cohort studies* 6

Case characteristics of women admitted in stable and critical conditions

Table S1: Case characteristics of women admitted in stable and critical conditions during the study period.

	Stable (N=262)	Critically ill (N=398)	Total (N=660)	P value†
**Antenatal care**				< 0·01
**N**	262	398	660	
Yes	233 (88·9%)	233 (58·5%)	466 (70·6%)	
No	29 (11·1%)	165 (41·5%)	194 (29·4%)	
**Danger signs**				0·21
**N**	233	233	466	
Yes	52 (22·3%)	43 (18·5%)	95 (20·4%)	
No	171 (73·4%)	172 (73·8%)	343 (73·6%)	
Other	10 (4·3%)	18 (7·7%)	28 (6·0%)	
**Partograph used**				< 0·01
**N**	195	245	440	
Yes	139 (71·3%)	95 (38·8%)	234 (53·2%)	
No	49 (25·1%)	66 (26·9%)	115 (26·1%)	
Unknown	7 (3·6%)	84 (34·3%)	91 (20·7%)	
**Labour started**				0·10
**N**	167	206	373	
Central hospital	15 (9·0%)	27 (13·1%)	42 (11·3%)	
Community/rural hospital	3 (1·8%)	9 (4·4%)	12 (3·2%)	
District/CHAM/Private hospital	45 (26·9%)	53 (25·7%)	98 (26·3%)	
Health center/Private clinic	18 (10·8%)	25 (12·1%)	43 (11·5%)	
Home	72 (43·1%)	63 (30·6%)	135 (36·2%)	
On the way/Before arrival to Health Facility	2 (1·2%)	1 (0·5%)	3 (0·8%)	
Other	12 (7·2%)	26 (12·6%)	38 (10·2%)	
Standalone maternity unit	0 (0·0%)	1 (0·5%)	1 (0·3%)	
TBA	0 (0·0%)	1 (0·5%)	1 (0·3%)	
**Ownership of Health Facility**				0·59

**N**	145	181	326	
CHAM	19 (13.1%)	35 (19.3%)	54 (16.6%)	
Local government	8 (5.5%)	10 (5.5%)	18 (5.5%)	
MoH	86 (59.3%)	94 (51.9%)	180 (55.2%)	
Private for profit	1 (0.7%)	1 (0.6%)	2 (0.6%)	
Other	31 (21.4%)	41 (22.7%)	72 (22.1%)	
**Delivered before death**				
**N**	262	396	658	< 0·01
Yes	197 (75·2%)	249 (62·9%)	476 (67·8%)	
No	65 (24·8%)	147 (37·1%)	212 (32·2%)	
**Mode of Delivery**				< 0·01
**N**	196	248	444	
Breech	4 (2·0%)	2 (0·8%)	6 (1·4%)	
Caesarean section	116 (59·2%)	109 (44·0%)	225 (50·7%)	
Destructive operation	2 (1·0%)	1 (0·4%)	3 (0·7%)	
Other	5 (2·6%)	7 (2·8%)	12 (2·7%)	
Spontaneous vaginal delivery	67 (34·2%)	125 (50·4%)	192 (43·2%)	
Vacuum	2 (1·0%)	4 (1·6%)	6 (1·4%)	
**Delivered by**				< 0·01
**N**	193	239	432	
Clinical officer	111 (57·5%)	70 (29·3%)	181 (41·9%)	
Medical officer	12 (6·2%)	31 (13·0%)	43 (10·0%)	
Midwife	63 (32·3%)	84 (35·3%)	147 (33·9%)	
Nurse	0 (0·0%)	2 (0·8%)	2 (0·5%)	
Obstetrician gynecologist	1 (0·5%)	2 (0·8%)	3 (0·7%)	
Other	6 (3·1%)	49 (20·5%)	55 (12·7%)	
**Condition at death**				< 0·01
**N**	262	398	660	
Abortion	9 (3·4%)	43 (10·8%)	52 (7·9%)	
During delivery	26 (9·9%)	14 (3·5%)	40 (6·1%)	
During pregnancy	43 (16·4%)	91 (22·9%)	134 (20·3%)	
Ectopic	1 (0·4%)	8 (2·0%)	9 (1·4%)	
Post-partum period (within 24hrs)	138 (52·7%)	91 (22·9%)	229 (34·7%)	
Post-partum period (after 24hrs but within 42 days)	45 (17·2%)	151 (7·9%)	196 (9·7%)	

† p-values for categorical variables were obtained through Fisher's exact tests.

Temporal changes in maternal mortality at the district level

The figure S1shows the temporal trends in maternal mortality at the district level. There were changes in the maternal mortality ratios between August 2020 – August 2022. Overall, MMR remained relatively high in most of the districts. It can also be noted that some districts did not report maternal deaths in some months during the two years.

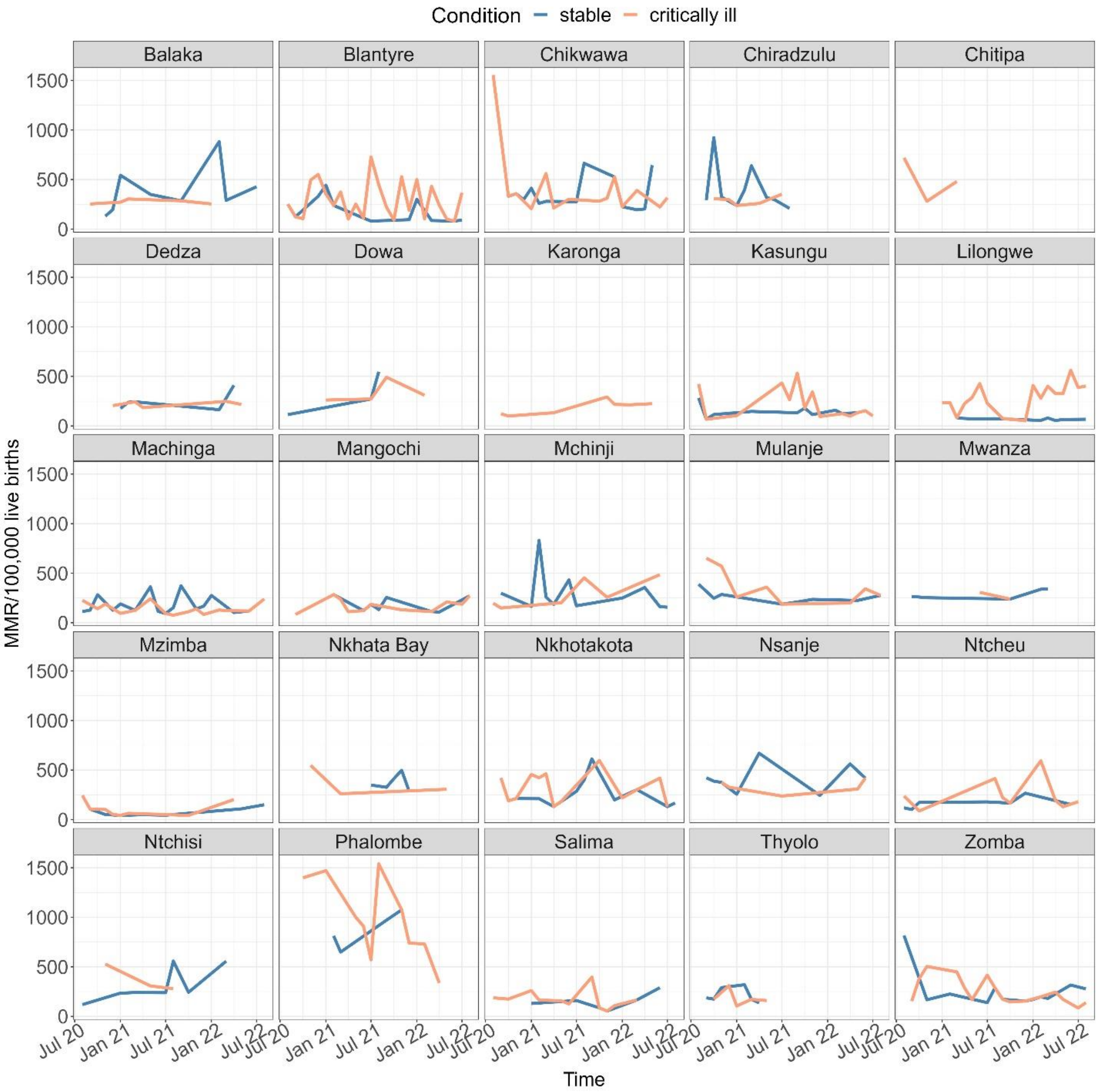

Figure S1: Temporal changes in maternal mortality by condition on admission, whether stable or critical, for all districts in Malawi. Likoma, Rumphi and Neno had single time points thereby not visible on the graph.

Principal component analysis

We reduced the dimensionality of the associated factors leading to mortality data to get more insight into a few variables likely to be strongly associated with the principal components. The first five principal components contributed 50% of the total variance (see figure S2)

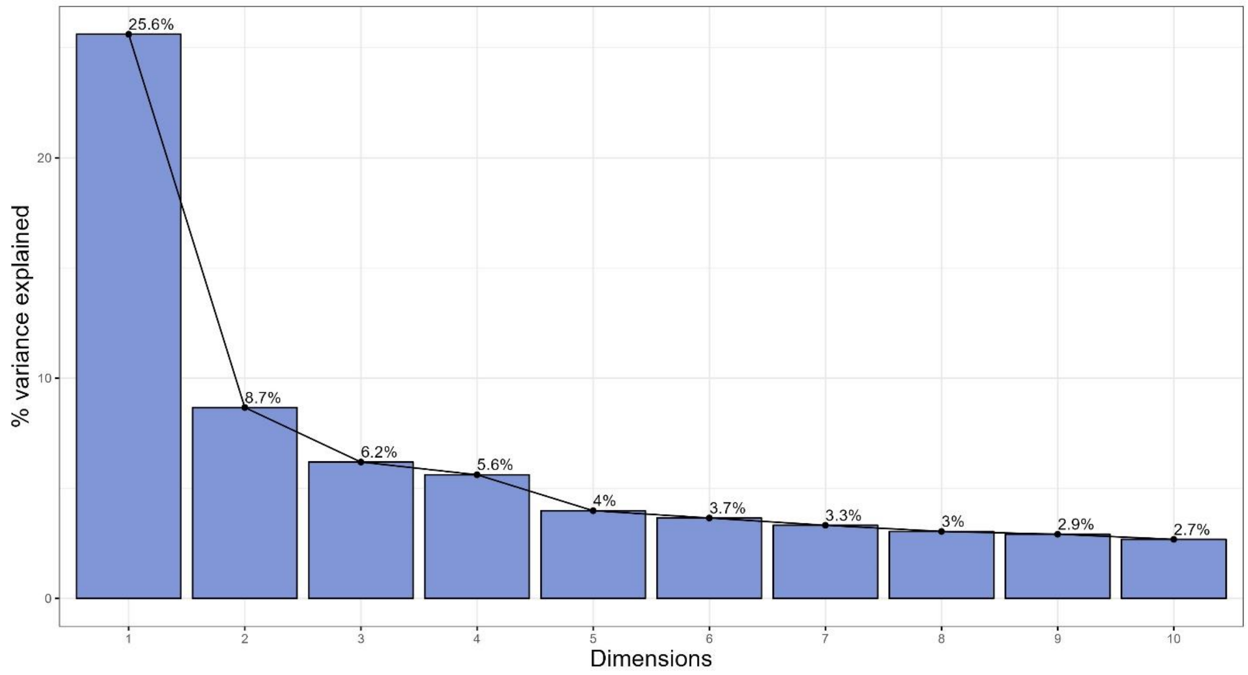

Figure S2: Principal component analysis of the associated factors leading to maternal death data. Scree plot showing the contribution of ten components to the total variance in the factors associated with maternal death. The components are presented in descending order. PC1 explains 25·6% of the variation while PC2 explains 8·7%, followed by PC3 at 6·2%. Cumulatively, the first five components explain 50% of the variance.

Selection of variables with a stronger contribution to principal components

After the PCA, we selected the first five dimensions that cumulatively explained 50% of the variance in the data. Assuming equal contribution by each variable, each variable would contribute 2.55% to each component. In this analysis, we set the contribution bar at 4% to get a stronger contribution.

Table S2: Influential variables under each principal component

PC1	Delay in receiving treatment
	Prolonged observation without action
	Inadequate resuscitation provided
	Non-avoidable factors
	Incomplete assessment
	Inadequate monitoring
	Lack of essential equipment
	Wrong treatment
PC2	
	Failure to accept limitations
	Delay in deciding to refer to facility (from home)
	Use of traditional medicine
	Failure to recognize danger
	Unsafe cultural practices

	Lack of transport
PC3	Unsafe self-medications
	Lack of antibiotics
	Unsafe cultural practices
	Non-avoidable factors
	Refusal of treatment
PC4	No information
	Inadequate midwifery skills
	Non-avoidable factors
	Lack of laboratory facilities
	Partograph incorrectly/not used
PC5	Lack of other essential drugs
	Communication problems between health facilities
	Lack of transport (from home)
	Uncertified providers

Table S3: Influential variables with at least 4% contribution to each of the selected five principal components. These factors were identified as contributing to maternal mortality for women admitted in stable and critical condition

Factor	Type of factor
Inadequate resuscitation	Administrative
Lack of essential equipment	Administrative
No information	Administrative
Lack of laboratory facilities	Administrative
Lack of antibiotics	Administrative
Lack of other essential obstetric drugs	Administrative
Communication problems between health facilities	Administrative
Prolonged abnormal observation without action	Health care worker
No avoidable factors	Health care worker
Incomplete assessment	Health care worker
Delay in deciding to refer (between health facilities)	Health care worker
Delay in treatment	Health care worker
Wrong treatment	Health care worker
Inadequate monitoring	Health care worker
Uncertified provider	Health care worker
Partograph incorrectly/not used	Health care worker
No treatment	Health care worker
Lack of obstetric lifesaving skills	Health care worker
Inadequate midwifery skills	Health care worker
Failure to recognize danger	Health care worker
Failure to accept limitations	TBA/community
Delay in deciding to refer (from community to health facility)	TBA/community
Delay reporting to health facility	Community
Unsafe trad/cultural practices	Community

Use of traditional medicine	Community
Unsafe self-medication	Patient/family
Refusal of treatment	Patient/family
Lack of transport from home to facility	Patient/family

STROBE Statement—Checklist of items that should be included in reports of *cohort studies*

	Item No	Recommendation	Page No
**Title and abstract**	1	(*a*) Indicate the study's design with a commonly used term in the title or the abstract<br>(*b*) Provide in the abstract an informative and balanced summary of what was done and what was found	2
**Introduction**			
Background/rationale	2	Explain the scientific background and rationale for the investigation being reported	3
Objectives	3	State specific objectives, including any prespecified hypotheses	3
**Methods**			
Study design	4	Present key elements of study design early in the paper	3
Setting	5	Describe the setting, locations, and relevant dates, including periods of recruitment, exposure, follow-up, and data collection	3
Participants	6	(*a*) Give the eligibility criteria, and the sources and methods of selection of participants. Describe methods of follow-up<br>(*b*) For matched studies, give matching criteria and number of exposed and unexposed	3
Variables	7	Clearly define all outcomes, exposures, predictors, potential confounders, and effect modifiers. Give diagnostic criteria, if applicable	3
Data sources/ measurement	8*	For each variable of interest, give sources of data and details of methods of assessment (measurement). Describe comparability of assessment methods if there is more than one group	3
Bias	9	Describe any efforts to address potential sources of bias	3,4
Study size	10	Explain how the study size was arrived at	3
Quantitative variables	11	Explain how quantitative variables were handled in the analyses. If applicable, describe which groupings were chosen and why	3
Statistical methods	12	(*a*) Describe all statistical methods, including those used to control for confounding<br>(*b*) Describe any methods used to examine subgroups and interactions<br>(*c*) Explain how missing data were addressed	3

		(*d*) If applicable, explain how loss to follow-up was addressed<br>(*e*) Describe any sensitivity analyses	
**Results**			
Participants	13*	(a) Report numbers of individuals at each stage of study—eg numbers potentially eligible, examined for eligibility, confirmed eligible, included in the study, completing follow-up, and analysed<br>(b) Give reasons for non-participation at each stage<br>(c) Consider use of a flow diagram	4
Descriptive data	14*	(a) Give characteristics of study participants (eg demographic, clinical, social) and information on exposures and potential confounders<br>(b) Indicate number of participants with missing data for each variable of interest<br>(c) Summarise follow-up time (eg, average and total amount)	4,5,6,7
Outcome data	15*	Report numbers of outcome events or summary measures over time	7

Main results	16	(*a*) Give unadjusted estimates and, if applicable, confounder-adjusted estimates and their precision (eg, 95% confidence interval). Make clear which confounders were adjusted for and why they were included<br><br>(*b*) Report category boundaries when continuous variables were categorized<br><br>(*c*) If relevant, consider translating estimates of relative risk into absolute risk for a meaningful time period	4,5,6,7,8
Other analyses	17	Report other analyses done—eg analyses of subgroups and interactions, and sensitivity analyses	NA
**Discussion**			
Key results	18	Summarise key results with reference to study objectives	8,9,10
Limitations	19	Discuss limitations of the study, taking into account sources of potential bias or imprecision. Discuss both direction and magnitude of any potential bias	10
Interpretation	20	Give a cautious overall interpretation of results considering objectives, limitations, multiplicity of analyses, results from similar studies, and other relevant evidence	10
Generalisability	21	Discuss the generalisability (external validity) of the study results	8,9,10
**Other information**			
Funding	22	Give the source of funding and the role of the funders for the present study and, if applicable, for the original study on which the present article is based	11

*Give information separately for exposed and unexposed groups.

Note: An Explanation and Elaboration article discusses each checklist item and gives methodological background and published examples of transparent reporting. The STROBE checklist is best used in conjunction with this article (freely available on the Web sites of PLoS Medicine at http://www.plosmedicine.org/, Annals of Internal Medicine at http://www.annals.org/, and Epidemiology at http://www.epidem.com/). Information on the STROBE Initiative is available at http://www.strobe-statement.org.
